# Basal keratinocyte tetrasomy in low-grade squamous intra-epithelial lesions of the cervix is restricted to high and intermediate risk HPV infection but is not type-specific

**DOI:** 10.1054/bjoc.1999.0937

**Published:** 2000-01-18

**Authors:** A Giannoudis, M F Evans, S A Southern, C S Herrington

**Affiliations:** Department of Pathology, University of Liverpool, Royal Liverpool University Hospital, Duncan Building, Daulby Street, Liverpool, L69 3GA, UK

**Keywords:** cervix, human papillomavirus, keratinocyte, chromosome

## Abstract

Human papillomavirus (HPV) infection appears to be an early event in cervical carcinogenesis with additional abnormalities being required for biological transformation. We have analysed 179 low-grade cervical squamous intra-epithelial lesions (SILs) and 15 normal cervices for the presence of HPV using both in situ hybridization and polymerase chain reaction (PCR). PCR was performed with GP5+/GP6+ primers followed by hybridization using probes for low (HPV 6, 11, 40, 42, 43, 44), intermediate (HPV 31, 33, 35, 39, 51, 52, 58, 59, 66 and 68) and high-risk HPVs (HPV 16, 18, 45 and 56). Interphase cytogenetic analysis using pericentromeric probes for chromosomes 1, 3, 4, 6, 10, 11, 17, 18 and X was also performed to identify numerical chromosomal abnormalities. Tetrasomy of all nine chromosomes was identified within basal keratinocytes, was restricted to epithelia infected with high risk (17 of 46) or intermediate risk (23 of 83) HPVs but was not HPV type-specific. Tetrasomy was not identified in any of the epithelia infected with low risk HPVs (*n* = 62). These numbers include multiple infection. These findings indicate that the induction of tetrasomy is a property restricted to high and intermediate-risk HPV types but that it is not type-specific. The factors governing which lesions will develop this abnormality are as yet unclear. © 2000 Cancer Research Campaign


*British Journal of Cancer* (2000) **82**(2), 424–428
© 2000 Cancer Research Campaign
Article no. bjoc.1999.0937

### Basal keratinocyte tetrasomy in low-grade squamous intra-epithelial lesions of the cervix is restricted to high and intermediate risk HPV infection but is not type-specific

**A Giannoudis, MF Evans, SA Southern and CS Herrington**

University of Liverpool, Department of Pathology, Royal Liverpool University Hospital, Duncan Building, Daulby Street, Liverpool L69 3GA, UK

**Summary** Human papillomavirus (HPV) infection appears to be an early event in cervical carcinogenesis with additional abnormalities being required for biological transformation. We have analysed 179 low-grade cervical squamous intra-epithelial lesions (SILs) and 15 normal cervices for the presence of HPV using both in situ hybridization and polymerase chain reaction (PCR). PCR was performed with GP5+/GP6+ primers followed by hybridization using probes for low (HPV 6, 11, 40, 42, 43, 44), intermediate (HPV 31, 33, 35, 39, 51, 52, 58, 59, 66 and 68) and high-risk HPVs (HPV 16, 18, 45 and 56). Interphase cytogenetic analysis using pericentromeric probes for chromosomes 1, 3, 4, 6, 10, 11, 17, 18 and X was also performed to identify numerical chromosomal abnormalities. Tetrasomy of all nine chromosomes was identified within basal keratinocytes, was restricted to epithelia infected with high risk (17 of 46) or intermediate risk (23 of 83) HPVs but was not HPV type-specific. Tetrasomy was not identified in any of the epithelia infected with low risk HPVs (*n* = 62). These numbers include multiple infection. These findings indicate that the induction of tetrasomy is a property restricted to high and intermediate-risk HPV types but that it is not type-specific. The factors governing which lesions will develop this abnormality are as yet unclear. © 2000 Cancer Research Campaign

**Keywords:** cervix; human papillomavirus; keratinocyte; chromosome

Human papillomaviruses (HPV) are members of a group of small, double-stranded DNA viruses which are causally associated with anogenital carcinomas and particularly with cervical carcinoma (Arends et al, 1990). The mechanisms by which the HPVs function in malignant progression appear predominantly to involve the activity of the two viral oncoproteins, E6 and E7. However, the development of invasive carcinoma is a multistep process involving the accumulation of a number of mutational events. Alterations to oncogenes and/or tumour suppressor genes, as well as parameters such as smoking and other viral infections appear to be required in parallel to infection (Herrington, 1995). In vitro studies have shown that HPV can induce genetic abnormalities including chromosomal imbalances in stably transfected cells (Hashida and Yasumoto, 1991; White et al, 1994; Steenbergen et al, 1996; Salinas-Toldo et al, 1997). However, stable transfection is associated with viral integration. Consequently, early genetic events related to productive infection cannot be studied by this approach. Moreover, although chromosome abnormalities have long been known to occur in invasive cervical carcinoma (Atkin, 1997), it has not been established at which stage of in vivo transformation these changes occur.

A preliminary study from our group showed that numerical chromosomal abnormalities occur in some HPV-infected low-grade cervical squamous intra-epithelial lesions (SILs) (Southern et al, 1997). In this study, we have extended this to a large series of low-grade SILs and have analysed a broader range of chromosomes by including chromosomes 4, 6 and 10 which have been reported to be abnormal in in vitro studies of HPV-induced immortalization (Steenbergen et al, 1996; Salinas-Toldo et al, 1997). Interphase cytogenetics showed tetrasomy with all of the chromosomes in 17/46 lesions infected with high-risk HPVs and 23/83 lesions infected with intermediate-risk HPVs: this difference was not statistically significant ($\chi^2$ = 1.18, df = 1, $P$ > 0.05). In all lesions, tetrasomy was present throughout the thickness of the epithelium, including the basal layer. None of the lesions infected with low-risk types or the 15 normal cervices showed any numerical chromosomal abnormality. Basal keratinocyte tetrasomy was therefore restricted to infection with high- and intermediate-risk HPVs. Although infection with a wide range of HPV types was identified, the presence of tetrasomy was not type-specific.

*Received 6 May 1999*
*Revised 21 July 1999*
*Accepted 3 August 1999*

*Correspondence to:* CS Herrington

### MATERIALS AND METHODS

### Tissue specimens

One hundred and seventy-nine low-grade squamous intra-epithelial lesions (SILs) and 15 normal cervices were identified from the diagnostic files of the Royal Liverpool University Hospital. The low-grade lesions comprised condylomata acuminata (*n* = 27), flat condylomata (*n* = 53) and cervical intra-epithelial neoplasia (CIN) grade 1 (*n* = 99). All of the diagnoses were confirmed by CSH.

### DNA extraction

Three 5-μm formalin-fixed, paraffin sections of cervical biopsy material were dewaxed in xylene (2 × 5 min) followed by 96%

ethanol (2 × 5 min), then pelleted, air-dried and incubated overnight at 42°C with 200 μg $ml^{-1}$ proteinase K (Boehringer Mannheim) in 200 mM Tris–HCI pH 8.0, 1 mM EDTA. The digest was heated to 95°C for 20 min to stop enzymatic activity, centrifuged and the supernatant used for polymerase chain reaction (PCR). The quality of the DNA was assessed by amplifying a 209 bp β-globin fragment using PCO3 and PCO5 primers (Jacobs et al, 1995).

### HPV typing by PCR

Ten to 15 μl of the extracted DNA was used in a 50 μl PCR reaction containing 50 mM potassium chloride, 10 mM Tris–HCI (pH 8.3), 200 μM of each dNTP, 3.5 mM magnesium chloride, 1 U of *Taq* DNA polymerase and 50 pmol each of the consensus GP5+/GP6+ primers. The primers amplify a fragment of 140–150 bp (depending on HPV type) from the L1 gene. PCR conditions comprised an initial denaturation step at 95°C for 4 min followed by 40 cycles of: denaturation at 94°C for 1 min, annealing at 40°C for 2 min, extension at 72°C for 1.5 min, followed by a final extension step at 72°C for 7 min to ensure complete extension of the amplified DNA. Ten microlitres of the PCR product were analysed on a 1.7% agarose gel stained with ethidium bromide. The gel was then denatured and blotted on to positively charged nylon (Southern blot). The remaining PCR product was diluted 1:10 and denatured for 10 min at 95°C. One microlitre of denatured DNA was then applied on to individual nylon memranes and placed under UV light for 3 min, followed by baking at 80°C for 1 h.

Membranes were placed into pre-hybridization buffer (0.02% sodium dodecyl sulphate (SDS), 1% blocking reagent (Boehringer Mannheim) in 2 × standerd saline citrate (SSC) for 1 h at 55°C and hybridized overnight at the same temperature using individual 5′ digoxigenin-labelled oligonucleotide probes specific for low-risk (HPV 6, 11, 40, 42, 43 and 44), intermediate-risk (HPV 31, 33, 35, 39, 51, 52, 58, 59, 66 and 68) and high-risk (HPV 16, 18, 45, and 56) HPV types (Jacobs et al, 1995). Hybridization was followed by washes in post-hybridization buffer (0.1% SDS in 2 × SSC) 3 × 10 min at 55°C and in blocking solution (0.5% Tween-20, 1% blocking reagent in 0.1 M Tris–HCl, pH 7.5) for 45 min at room temperature. Signal was detected with anti-digoxigenin–alkaline phosphatase conjugate diluted 1:5000 in blocking solution for 30 min and developed using nitroblue tetrazolium/bromochloroindolyl phosphate (NBT/BCIP).

### HPV localization by in situ hybridization

Tissue sections were dewaxed in xylene, rehydrated through graded alcohols and washed in Molecular Biology Grade (MBG) water. Sections were digested with 0.5 mg $ml^{-1}$ proteinase K in 0.1 M Tris–HCl pH 7.6 for 20 min at 37°C and hybridized with a mixture of nick-translated digoxigenin labelled probes derived from HPV 6, 11, 16, 18, 31, 33 plasmid clones in 50% formamide, 20 × SSC, 10% dextran sulphate, under low stringency conditions to allow hybridization to a wide range of HPV types (Southern et al, 1997). When multiple infection was identified by PCR in lesions showing chromosome abnormalities, in situ hybridization with the appropriate individual probes was carried out to establish the morphological localization of the individual HPV types (i.e. HPV 6, 11, 16, 18, 31, 35, 45, 51, 56 and 66; see Results). All of the probes used for in situ hybridization consisted of the cloned whole viral genome (see Southern et al (1997) for details of the source of each clone).

### Interphase cytogenetic analysis

Tissue sections were dewaxed in xylene, rehydrated through graded alcohols, washed in MBG water and immersed in 1 M sodium thiocyanate at 80°C for 10 min. Sections were digested with 4 mg $ml^{-1}$ of pepsin (Sigma) in 0.2 M hydrochloric acid for 20 min at 37°C, followed by washes with water and air-drying. Chromosome probes (1, 3, 4, 6, 10, 11, 17, 18 X) (Oncor) at a concentration of 1 ng $\mu l^{-1}$ in 60% formamide, 2 × SSC, 10% dextran sulphate and 1 μg $\mu l^{-1}$ sheared salmon sperm DNA were applied to the dry sections. The probe and tissue were denatured at 80°C for 8 min and hybridized overnight in a 37°C oven, as previously described (Southern and Herrington, 1996). These probes allow the assessment of centromere number per cell nucleus but do not provide information about non-centromeric chromosomal areas. The number of signals per nucleus was counted in a total of 200 nuclei from each lesion following the rules previously established for signal counting. The presence of three and four signals per nucleus in > 10% of nuclei determines trisomic and tetrasomic cell populations respectively as previously validated (Southern and Herrington, 1996, 1997).

### RESULTS

### HPV typing and localization of infection

#### *HPV typing by PCR*

All of the tissues examined were positive for β-globin by PCR indicating the presence of adequate DNA for further analysis. One hundred and seventy-three of 179 specimens were found to be HPV-positive. Sixty-two lesions contained low-risk HPVs (HPV 6 ($n$ = 30), 11 ($n$ = 19), 40 ($n$ = 1), 42 ($n$ = 5), 43 ($n$ = 6), 44 ($n$ = 1)), 46 lesions contained high-risk HPVs (HPV 16 ($n$ = 21), 18 ($n$ = 13), 45 ($n$ = 4), 56 ($n$ = 8)) and 83 lesions contained intermediate-risk HPVs (HPV 30 ($n$ = 1), 31 ($n$ = 7), 33 ($n$ = 3), 35 ($n$ = 16), 39 ($n$ = 3), 51 ($n$ = 12), 52 ($n$ = 3), 58 ($n$ = 14), 59 ($n$ = 3), 66 ($n$ = 19), 68 ($n$ = 2)). HPV 30 and an unknown type with homology to HPV43 (77%), thus classified as low risk, were identified by DNA sequencing. Multiple types were identified in 17 cases (6/11, 6/16, 6/18, 6/31, 6/35 ($n$ = 2), 6/66, 11/18, 11/66, 16/56, 18/31, 18/56, 35/51, 35/66, 45/51, 51/66, and 59/51/42) and were included individually in the above HPV numbers. The 15 normal cervices were HPV-negative.

#### *In situ hybridization*

Six of the 173 specimens that were HPV-positive by PCR were negative by in situ hybridization. All the other specimens were positive with both methods allowing morphological localization of the infection to be established (Figure 1). The six non-infected cases and the 15 normal cervices were negative with both methods. In situ hybridization also allowed the localization of individual HPV types to different morphological areas in the nine biopsies in which both multiple HPV types and chromosome abnormalities were identified: this allowed accurate karyotypic assessment of these areas.

© 2000 Cancer Research Campaign 

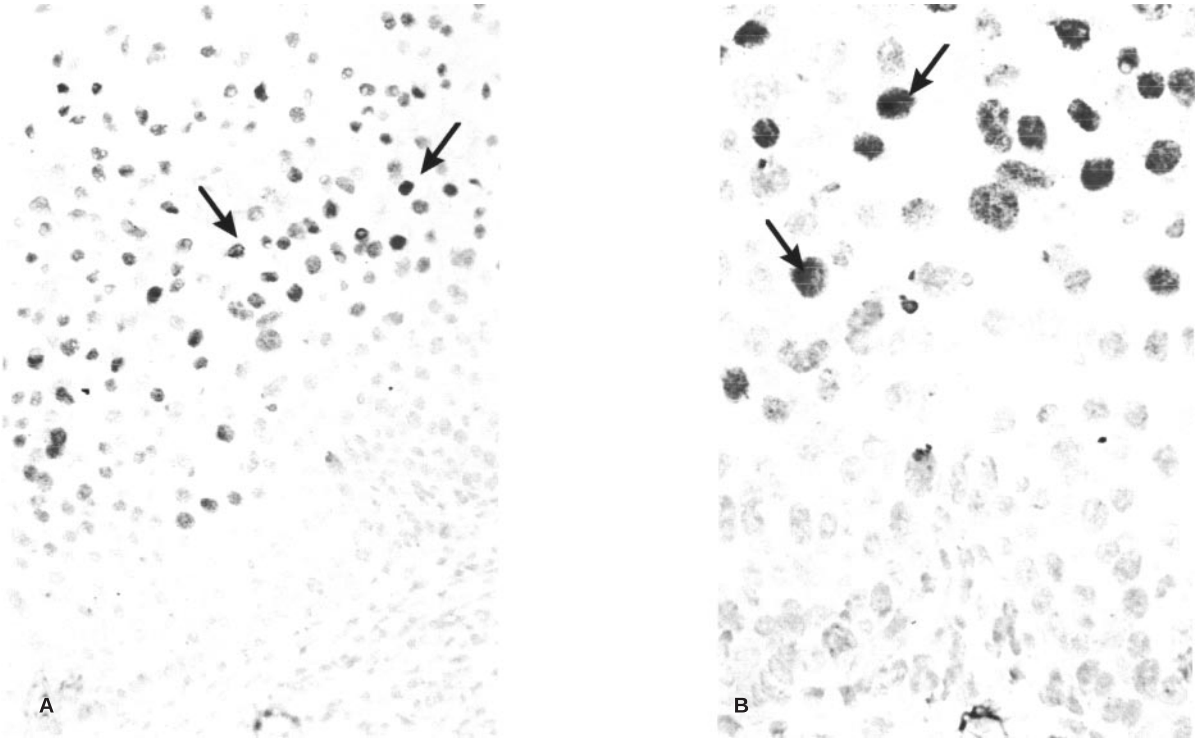

**Figure 1** A representative example of in situ hybridization of CIN 1 lesions using a cocktail of HPV probes. The arrows indicate HPV-infected cells. HPV typing by GP5+/GP6+ PCR revealed infection with HPV 66 in **A** and HPV 18 in **B**.

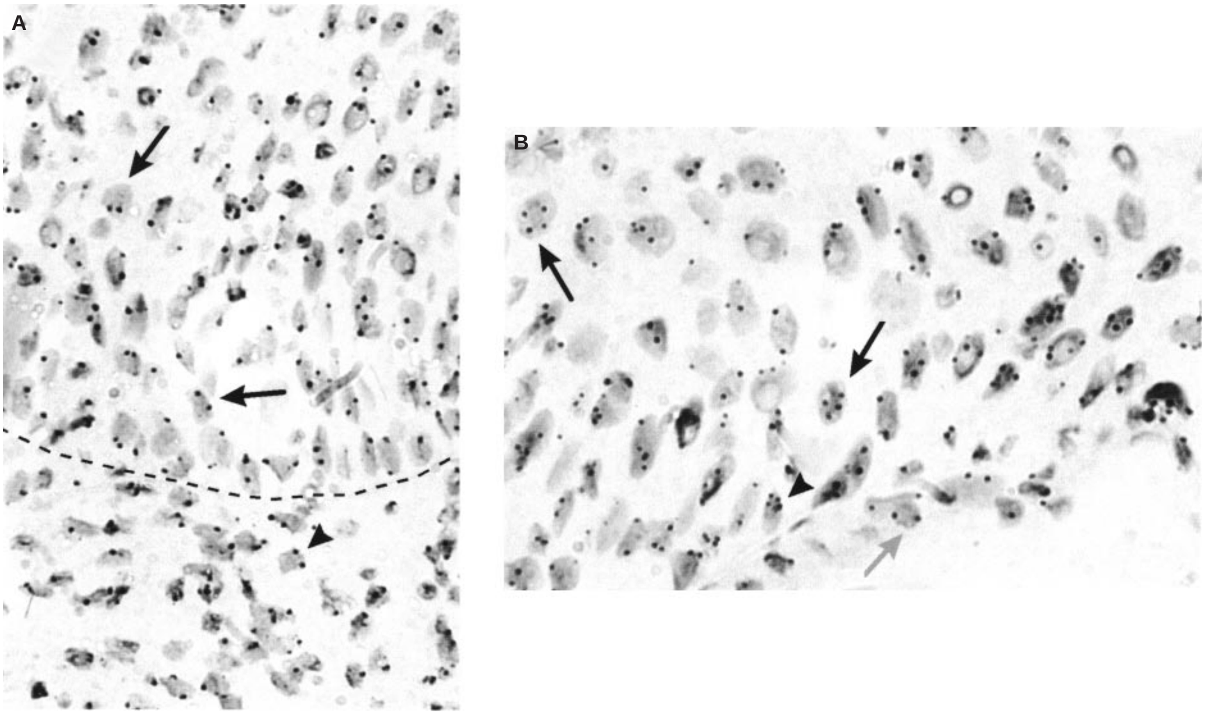

**Figure 2** Representative examples of interphase cytogenetic analysis using a centromeric probe for chromosome 1. Note the presence of two chromosome 1 signals in both epithelial (arrows) and stromal (arrowhead) cell nuclei in **A**. This contrasts with tetrasomy (four chromosome 1 signals) in epithelial cell nuclei (arrows), including basal keratinocytes (arrowhead), despite disomy of stromal cells (grey arrow) in **B**. The approximate position of the basement membrane is shown as a dotted line in **A**.

*© 2000 Cancer Research Campaign*

**Table 1** Association of numerical chromosomal abnormalities with HPV types. For the multiple infections showing tetrasomy, the HPV type present in the tetrasomic area is underlined

| HPV type | Disomy | Basal keratinocyte tetrasomy |
|---|---|---|
| Low risk | 51 | 0 |
| Intermediate risk | 51 | 17[a] |
| High risk | 23 | 14[b] |
| Multiple infections | 8[c] | 9[d] |

[a]Lesions containing HPV 31 (*n* = 2), 33 (*n* = 2), 35 (*n* = 1), 39 (*n* = 1), 52 (*n* = 1), 58 (*n* = 6), 66 (*n* = 4). [b]Lesions containing HPV 16 (*n* = 7), 18 (*n* = 6), 56 (*n* = 1). [c]Lesions containing HPV 6&11; 51&66; 35&66; 6&35; 6&31; 11&18; 6&18; 42,51&59. [d]Lesions containing HPV 6&66; 35&51; 18&56; 45&51; 16&56; 11&66; 6&35; 18&31; 6&16.

### Chromosome analysis

### *Correlation of tetrasomy with HPV types*

Tetrasomy was present with all nine chromosomal probes in a total of 40 lesions (Figure 2). In 36 lesions, tetrasomy persisted throughout the whole epithelial thickness, extending from basal to superficial epithelial cells. In four cases, initial sections showed apparently focal tetrasomy in suprabasal cells. Further sections were analysed from these lesions and tetrasomy of basal cells was identified indicating that the apparent suprabasal localisation of tetrasomy in the initial sections was due to cross-cutting artefact in small lesions. All the other lesions, including the normal control tissues were disomic, showing three signals in 0.5–2.5% of nuclei and four signals in 0.5%.

Overall, of the 46 lesions infected with high-risk HPVs, 17 showed basal cell tetrasomy and, of the 83 lesions infected with intermediate-risk HPVs, 23 showed basal cell tetrasomy (Figure 2). The difference between these two groups is not statistically significant ($\chi^2 = 1.18$, df = 1, $P > 0.05$). All of the 62 lesions infected with low-risk HPVs were disomic. These figures include the multiple infections. Nine lesions showing basal cell tetrasomy were infected with more than one HPV type. In order to identify which HPV type was present in the area showing chromosome abnormality, in situ hybridization was performed using digoxigenin-labelled probes specific for each type. The results are summarized in Table 1.

Correlation of the interphase cytogenetic and HPV in situ hybridization data demonstrated that, although the chromosome abnormalities were confined to HPV-infected areas, they were not present throughout the HPV-infected area.

### DISCUSSION

In this study, we have demonstrated, in a series of 179 low-grade SILs of the cervix, that basal keratinocyte tetrasomy is associated with infection with high- and intermediate- but not low-risk HPV types. This is consistent with the fact that the E6 and E7 proteins of high-risk HPVs interact with components of the cell cycle control machinery, including p53, pRb and p21 (Slebos et al, 1994; Galloway and McDougall, 1996; Xiong et al, 1996). Endoreduplication has been described in association with the abrogation of p21 function as a result of E7 binding (Stewart et al, 1999). Similarly, duplication of cellular DNA content as identified by flow cytometry has been induced by E6, E7 and E2 protein expression, particularly in the presence of mitotic spindle inhibitors (di Leonardo et al, 1997; Frattini et al, 1997). These in vitro findings are consistent with the observation that mitotic checkpoint abnormalities can be induced by both E6 and E7 proteins (Steinmann et al, 1994; Thomas and Laimans, 1998). In addition, it has been shown that the absence or loss of function of wild-type p53 from mouse embryonic fibroblasts results in multiple copies of functionally competent centrosomes (Fukasawa et al, 1996). However, our finding of tetrasomy indicates not only chromosome re-duplication, consistent with endo-reduplication, but also centromere separation, implying that the mechanism by which cellular DNA is reduplicated involves separation of sister chromatids.

Although tetrasomy was only found in lesions infected with high- and intermediate-risk HPVs, it was not type-specific and there was no significant difference in the frequency of tetrasomy in lesions infected with intermediate-risk, by comparison with high-risk types. Whilst this observation casts some doubt over the distinction between high- and intermediate-risk HPVs, these two groups were defined on the basis of clinical risk association (Lorincz et al, 1992). These clinical associations are likely to be dependent upon a wide range of biological properties of the individual viral types and hence retention of the distinction between high- and intermediate-risk HPVs may be useful in identifying factors which are important in neoplastic transformation by these viruses. A wide range of HPV types was identified in the lesions analysed and tetrasomy was present in lesions infected with HPVs 16, 18, 31, 33, 35, 39, 51, 52, 56, 58 and 66. However, these viral types were also present in lesions showing no numerical chromosomal abnormality. In addition, HPV in situ hybridization demonstrated that basal cell tetrasomy was not present throughout the HPV-infected areas in any of the lesions showing tetrasomy. These two observations indicate that other factors are involved in determining whether basal cell tetrasomy will develop. Possible viral factors include: the level of viral early gene expression and its relationship to keratinocyte differentiation; the presence of different HPV-type variants; and persistence of viral infection, which has been shown to be an important factor in determining progression of intra-epithelial lesions (Remminck et al, 1995). Furthermore, cellular factors such as activation of oncogenes, inactivation of tumour suppressor genes, abnormalities of cell cycle regulators, smoking, immunosuppression and the presence of other infectious agents may also be involved (Herrington, 1995; Howley, 1996).

The morphological location of the numerical chromosomal abnormalities identified in this study suggests that the induction of tetrasomy is more likely to be related to initial viral early gene expression than to viral DNA replication. Tetrasomy was present in the basal and suprabasal cell layer and, in the majority of lesions, extends throughout the epithelium indicating persistence despite squamous cell differentiation. In four lesions, tetrasomy was present only in basal and suprabasal keratinocytes and did not extend to the superficial layers. This observation suggests that tetrasomy begins in the proliferating part of the epithelium and implies that this phenomenon is not directly related to viral DNA replication which occurs in the intermediate and superficial layers of the epithelium (Howley, 1996). Moreover, as it is known that the E6 and E7 genes are expressed in basal epithelial cells (Higgins et al, 1992; Nilsson et al, 1996), it is consistent with in vitro evidence that the E6 and E7 proteins can induce DNA re-replication and, after more prolonged exposure, chromosome abnormalities in transfected cells (Xiong et al, 1996).

© 2000 Cancer Research Campaign 

In conclusion, basal cell tetrasomy occurs in approximately 31% of low-grade cervical SILs infected with high- and intermediate-risk HPVs but not in those infected with low-risk HPVs. Tetrasomy is not type-specific and is not always present throughout the HPV-infected epithelium. Further investigation of this phenomenon will provide important information regarding the mechanisms by which HPVs interfere with cell cycle control.

### ACKNOWLEDGEMENTS

We thank the University of Liverpool for financial support.

### REFERENCES

Arends MJ, Wyllie AH and Bird CC (1990) Papillomaviruses and human cancer. *Hum Pathol* **21:** 686–698

Atkin NB (1997) Cytogenetics of carcinoma of the cervix uteri: a review. *Cytogenet Cell Genet* **95:** 33–39

di Leonardo A, Khan SH, Linke SP, Greco V, Seidita G and Wahl GM (1997) DNA rereplication in the presence of mitotic spindle inhibitors in human and mouse fibroblasts lacking either p53 or pRb function. *Cancer Res* **57**: 1013–1019

Frattini MG, Lim HB, Doorbar J and Laimins LA (1997) Induction of human papillomavirus type 18 late gene expression and genomic amplification in organotypic cultures from transfected DNA templates. *J Virol* **71**: 7068–7072

Fukasawa K, Choi T, Kuriyama R, Rulong S and Vande-Woude GF (1996) Abnormal centrosome amplification in the absence of p53. *Science* **271**: 1744–1747

Galloway DA and McDougall JK (1996) The disruption of cell cycle checkpoints by papillomavirus oncoproteins contributes to anogenital neoplasia. *Semin Cancer Biol* **7**: 309–315

Hashida T and Yasumoto S (1991) Induction of chromosomal abnormalities in mouse and human epidermal keratinocytes by the HPV type 16 E7 oncogene. *J Gen Virol* **72**: 1569–1577

Herrington CS (1995) Human papillomavirus and cervical neoplasia II: interaction with other factors. *J Clin Pathol* **48**: 1–6

Higgins GD, Uzelin M, Phillips GE, McEvoy P, Marin R and Burrell CJ (1992) Transcription patterns of human papillomavirus type 16 in genital intraepithelial neoplasia: evidence for promoter usage within the E7 open reading frame during epithelial differentiation. *J Gen Virol* **73**: 2047–2057

Howley PM (1996) Papillomaviridae: the viruses and their replication. In: *Fundamental Virology*, 3rd edn, Fields BN, Knipe DM and Howley PM (eds). Lippincott-Raven Publishers: Philadelphia

Jacobs MV, de Roda Husman AM, van den Brule AJC, Snijders PJF, Meijer CJLM and Walboomers JMM (1995) Group-specific differentiation between high and low risk human papillomavirus genotypes by general primer mediated PCR and two cocktails of oligonucleotide probes. *J Clin Microbiol* **33**: 901–905

Lorincz AT, Reid R, Jenson AB, Greenberg MD, Lancaster W and Kurman RJ (1992) Human papillomavirus infection of the cervix: relative risk associations of 15 common anogenital types. *Obstet Gynecol* **79**: 328–337

Nilsson CH, Bakos E, Petry KU, Schneider A and Dürst M (1996) Promoter usage in the E7 ORF of HPV 16 correlates with epithelial differentiation and is largely confined to low-grade genital neoplasia. *Int J Cancer* **65**: 6–12

Remmink AJ, Walboomers JMM, Helmerhorst TJM, Voorhorst FJ, Rozendaal L, Risse EKJ, Meijer CJLM and Kenemans P (1995) The presence of persistent high-risk HPV genotypes in dysplastic cervical lesions is associated with progressive disease – natural history up to 36 months. *Int J Cancer* **61**: 306–311

Salinas-Toldo S, Dürst M and Lichter P (1997) Specific chromosomal imbalances in human papillomavirus transfected cells during progression towards immortality. *Proc Natl Acad Sci USA* **94**: 3854–3859

Slebos RJC, Lee MH, Plunkett BS, Kessis TD, Williams BO, Jacks T, Hedrick L, Kastan MB and Cho KR (1994) p53 dependent G1 arrest involves pRb related proteins and is disrupted by the human papillomavirus 16 E7 oncoprotein. *Proc Natl Acad Sci USA* **91**: 5320–5324

Southern S and Herrington CS (1996) The assessment of intra-tumooural karyotypic heterogeneity by interphase cytogenetics in paraffin wax sections. *J Clin Pathol: Mol Pathol* **49**: M283–M289

Southern SA and Herrington CS (1997) Interphase karyotypic analysis of chromosomes 11, 17 and X in invasive squamous cell carcinoma of the cervix: morphological correlation with HPV infection. *Int J Cancer* **70**: 502–507

Southern SA, Evans MF and Herrington CS (1997) Basal cell tetrasomy in low grade cervical squamous intraepithelial lesions infected with high risk human papillomaviruses. *Cancer Res* **57**: 4210–4213

Steenbergen RDM, Walboomers JMM, Meijer CJLM, van der Raaij-Helmer EMH, Parker JN, Chow LT, Broker TK and Snijers PJF (1996) Transition of human papillomavirus type 16 and 18 transfected human foreskin keratinocytes towards immortality: activation of telomerase and allele losses at 3p, 10p, 11q and/or 18q. *Oncogene* **13**: 1249–1257

Steinmann KE, Pei XF, Stoppler H, Schlegel R and Schlegel R (1994) Elevated expression and activity of mitotic regulatory proteins in human papillomavirus-immortalised keratinocytes. *Oncogene* **9**: 387–394

Stewart ZA, Leach SD and Pietenpol JA (1999) p21Waf/Cip inhibition of cyclin E/Cdk2 prevents endoreduplication after mitotic spindle disruption. *Mol Cell Biol* **19**: 205–215

Thomas JT and Laimins LA (1998) Human papillomavirus oncoproteins E6 and E7 independently abrogate the mitotic spindle checkpoint. *J Virol* **72**: 1131–1137

White AE, Livanos EM and Tlsty TD (1994) Differential disruption of genomic integrity and cell cycle regulation in normal human fibroblasts by the HPV oncoproteins. *Genes Dev* **8**: 668–677

Xiong Y, Kuppuswamy D, Li Y, Livanos EM, Hixon M, White A, Beach D and Tlsty TD (1996) Alteration of cell cycle kinase complexes in human papillomavirus E6 and E7 expressing fibroblasts precedes neoplastic transformation. *J Virol* **70**: 999–1008

 © 2000 Cancer Research Campaign
